# Intrathecal T‐cell clonal expansions in patients with multiple sclerosis

**DOI:** 10.1002/acn3.310

**Published:** 2016-04-20

**Authors:** Alessandra de Paula Alves Sousa, Kory R. Johnson, Richard Nicholas, Sam Darko, David A. Price, Daniel C. Douek, Steven Jacobson, Paolo A. Muraro

**Affiliations:** ^1^ Neuroimmunology BranchViral Immunology SectionNational Institute of Neurological Disorders and StrokeNIHBethesdaMaryland; ^2^ Division of Brain SciencesFaculty of MedicineImperial College LondonLondonUnited Kingdom; ^3^ Bioinformatics SectionNational Institute of Neurological Disorders and StrokeNIHBethesdaMaryland; ^4^ Imperial College Healthcare NHS TrustLondonUnited Kingdom; ^5^ Human Immunology SectionVaccine Research CenterNational Institute of Allergy and Infectious DiseasesNIHBethesdaMaryland; ^6^ Institute of Infection and ImmunityCardiff University School of MedicineCardiffUnited Kingdom

## Abstract

**Objective:**

Analysis of the T‐cell receptor (TCR) repertoire in the cerebrospinal fluid (CSF) of patients with multiple sclerosis (MS) can reveal antigen‐specific immune responses potentially implicated in the disease process. We applied a new unbiased deep‐sequencing method for TCR repertoire analysis to accurately measure and compare receptor diversity and clonal expansions within the peripheral and CSF‐trafficking T‐cell populations of patients with MS and control individuals with idiopathic intracranial hypertension (IIH).

**Methods:**

Paired blood and CSF TCR *β*‐chain libraries from five MS patients and five IIH controls were sequenced, yielding a total of 80 million reads.

**Results:**

Although TCR repertoire diversity was greater in the blood and CSF compartments of MS patients when compared with IIH controls, it is notable that the frequency of clonal expansions was also significantly higher in both compartments of MS patients. Highly expanded T‐cell clones were enriched in the CSF compartment of MS patients compared to peripheral blood, very few of them were detected in both compartments.

**Interpretation:**

Collectively, our data provide a proof of principle that private compartmentalized T‐cell expansion exists in the intrathecal space of MS patients.

## Introduction

Multiple sclerosis (MS) is a neuroinflammatory disorder that affects over 2.5 million people worldwide. The occurrence of clinical relapses and inflammatory lesions in MS patients is associated with the infiltration of T cells in the central nervous system (CNS), which potentially react with myelin proteins and drive the inflammatory and demyelinating process.^1^ Although the etiology of MS remains unknown, a number of environmental factors including viral infection (e.g., Epstein–Barr virus),^2^ vitamin D deficiency, and smoking may lead to a dysregulated adaptive immunity in genetically susceptible individuals.^3^ Treatments for MS have primarily involved modulation or suppression of the immune response.^4^

T‐cell receptor (TCR) complementary‐determining region 3 (CDR3) length spectratyping has been used as a tool to identify skewing of the TCR repertoire in the peripheral blood (PB) of patients with MS,^5^ as well as in brain‐infiltrating T cells and the cerebrospinal fluid (CSF) compartment.^6^, ^7^ In one study, the expansion of immunodominant clones in the CSF compartment correlated with neurological exacerbation and the TCR repertoire normalized with clinical remission.^8^ Sequence analysis in CD4^+^ and CD8^+^ T cells infiltrating actively demyelinating MS lesions reveals that the majority of CD4^+^ T‐cell population was more heterogeneous compared to CD8^+^ T cells, which were represented in 35% of total clones in one MS case.^9^ However, spectratyping and conventional sequencing methods do not allow sufficient throughput to sequence large numbers of TCRs and reliably characterize the repertoire. The advent of deep sequencing and its application to analysis of the TCR repertoire has now enabled the accurate identification and quantification of individual clones in complex polyclonal populations.^10^

To interrogate the peripheral and CSF T‐cell repertoire in MS patients, we applied an unbiased method to amplify and deep sequence TCR *β*‐chain rearrangements present in unsorted and nonstimulated cells from these compartments. As controls, samples were also processed from individuals with idiopathic intracranial hypertension (IIH), a disorder characterized by increased intracranial pressure of unknown cause.^11^ Significantly enriched high‐frequency clones were present in the CSF of MS patients, suggesting a compartmentalized expansion of disease‐relevant T cells. By contrast, the number of high‐frequency clones found in the CSF of controls was not different from the PB compartment.

## Methods

### Patients and sample collection

Paired PB and CSF samples were collected from five relapsing–remitting MS patients and five IIH controls. All patients were assessed under the care of the Neurology Unit at Charing Cross Hospital, Imperial College Healthcare NHS Trust, London, UK. None of the MS patients included in this study was receiving any form of immunotherapy at the time of sample collection. Peripheral blood mononuclear cells (PBMCs) were purified by density gradient centrifugation (Ficoll‐HyPaque^TM^Plus; Amersham Bioscience, Piscataway, NJ, USA) and a total of 10^6^ cells was stored in RNA*later* ^TM^ solution (Ambion, Waltham, MA, USA) at −80°C until RNA extraction. All CSF samples were obtained via nontraumatic collection assessed at the time of lumbar puncture by visual inspection and microscopic examination. CSF cells were also cryopreserved in RNA*later* ^TM^ solution.

### TCRβ library preparation

The V‐CDR3‐J region of the TCR*β* chain was amplified from unsorted PBMCs (1 × 10^6^) and CSF cells (3.7 × 10^4^ to 2.2 × 10^5^) isolated from MS patients and IIH controls. The TCR*β* library generated for high‐throughput DNA sequencing was prepared according to a previously published protocol with minor modifications.^12^ Briefly, mRNA was extracted using immunomagnetic columns (Miltenyi Biotec, Germany) following the manufacturer's protocol. cDNA was generated using a template‐switch anchored reverse‐transcription polymerase chain reaction (RT‐PCR) based on the SMARTer pico cDNA PCR Synthesis Kit (Clontech, Mountain View, CA, USA). The final cDNA product was then subjected to a clean‐up step using PCR columns (Nucleospin II; Macherey‐Nagel, Germany). To amplify the V‐CDR3‐J region of the TCR*β* chain, a nested PCR was performed using a pair of primers, one binding the SMARTer oligonucleotide region (5’‐AAGCAGTGGTATCAACGCAGAGTAC‐3’), and the other binding the TCR*β* constant region (5’‐TGCTTCTGATGGCTCAAACACAGCGACCT‐3’). The qPCR assays were run using the Kapa HiFi system (Kapa Biosystems, Wilmington, MA, USA) as follows: one cycle at 95°C for 5 min; five cycles at 98°C for 20 sec and 72°C for 1 min; and 35 cycles at 98°C for 20 sec and 68°C for 1 min. TCR*β* DNA library from each sample was purified using the e‐Gel 2% agarose system (Invitrogen, USA) and a unique band of ≅650 bp size was collected into a well filled with *RNase/DNase*‐free water. A second PCR was performed to add an Illumina adaptor and a unique index barcode to each individual sample. Library size and quality were measured using an Agilent Bioanalyzer High Sensitivity DNA Chip (Agilent, USA) and library concentration was calculated using Kapa Illumina Library Quantification Assay (Kapa Biosystems, Santa Clara, CA, USA).

### High‐throughput sequencing and bioinformatics analysis

High‐throughput sequencing was performed by the Genomic Core Facility of the Medical Research Center (MRC) at Imperial College London. A total of 2 nmol of each sample (*n* = 20) was pooled for high‐throughput sequencing on the HiSeq 2500 platform (Illumina, USA). A total of 80‐million paired TCR*β* sequences consisting of 150 bases at 5’ forward and 3’ reverse orientation were generated. This sequencing protocol was developed and validated as described previously.^12^ Read 1 (5’ forward orientation) was sequenced using the designed Illumina primer, whereas read 2 (3’ reverse orientation) was sequenced using a pool of 13 primers binding the TCR*β* J region at a final concentration of 100 *μ*M each (Invitrogen, USA). TCR*β* annotation was performed by combining a custom Java program written in‐house and the BLAST program developed by the National Center for Biotechnology Information. Briefly, the V and J germline genes of a TCR*β* read were identified first, and the CDR3 was determined by finding the conserved cysteine at 5’ end and the conserved phenylalanine at the 3’ end. Unique and productive TCR*β* combinations (V‐D‐J) were collapsed to determine the count (TCR in‐frame reads). For each combination, the number of nucleotides contributed by the germline V, D, and J genes was determined together with the number of nucleotide additions. Shannon entropy (diversity), species richness, and evenness were calculated for each TCR*β* repertoire by using the R package, Vegan. Entropy and richness were normalized by calculating the maximum Shannon entropy and maximum combination richness for each repertoire based on the cell count used for library generation. The calculated Shannon entropy and combination richness were then divided by the respective maxima to return a number between 0.0 and 1.0. For each repertoire, the average and standard deviation were calculated for germline index, CDR3 length, and number of nucleotide additions. Custom Perl scripts were used to calculate the distribution of CDR3 length, V‐J pairing percentage, and amino acid compositions of each CDR3 position, as well as whole CDR3s from all of the annotated TCR*β* sequence reads in each subject and group.

### Statistics

**Figure 1:**
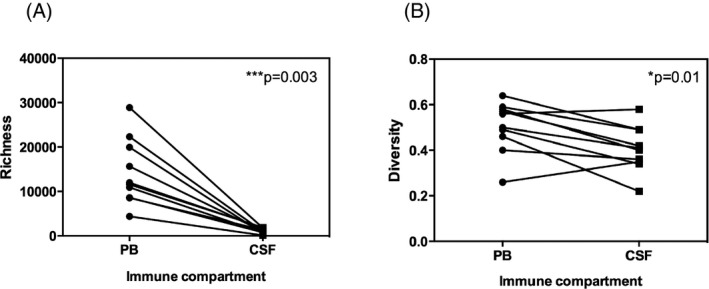
Richness and diversity of the T‐cell receptor (TCR) *β* repertoire in the peripheral blood and cerebrospinal fluid (CSF). TCR *β* richness (number of unique TCR *β* clonotypes) was significantly greater in the peripheral blood (PB) than in the CSF for all subjects. The unpaired nonparametric T‐test was applied with *p*‐value 0.0039 (**A**). TCR *β* diversity (number of unique TCR *β* clonotypes per sample normalized by cell count) was also cumulatively higher in PB compared with CSF. LOO *p*‐value test ranged 0.0078–0.027 (**B**). LOO, Leave‐One‐Out.

**Figure 2:**
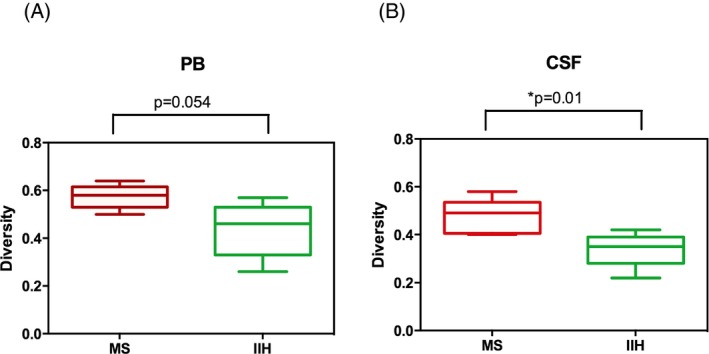
Diversity of the TCR *β* repertoire in the peripheral blood and cerebrospinal fluid (CSF) of multiple sclerosis (MS) patients and idiopathic intracranial hypertension (IIH) controls. A significantly higher diversity was detected in the PB (**A**) Leave‐One‐Out (LOO *p*‐value test ranged 0.015–0.063) and CSF compartment (**B**) (LOO *p*‐value test ranged 0.019–0.063) of MS patients compared to IIH controls. PB, peripheral blood. TCR, T‐cell receptor.

All statistical analyses were performed in Prism (http://www.graphpad.com/scientific-software/prism/) or R (http://www.r-project.org/). Clone frequencies observed in each subject (http://data.ninds.nih.gov/Jacobson/Alessandra/index.html) were first converted to a percent total frequency value by dividing each clone frequency by the cumulative frequency observed across all clones for the same sample. Degree of expansion per clone was then calculated by taking the Log2 transform of the percent total frequency value. To compare richness, diversity, and clone expansion between sample classes (i.e., MS and IIH), the unpaired nonparametric Wilcoxon signed rank T‐test was used (*α* = 0.05). To compare expansion for clones observed across compartments (i.e., PB and CSF), the paired nonparametric Wilcoxon signed rank sum test was used (*α* = 0.05). In addition, we also calculated the *p*‐value by applying the Leave‐One‐Out (LOO) test on the Figures 1A‐B, 2A‐B.

By applying the phylogenetic tree analysis, alignment of CDR3 amino acid sequences from shared clonotypes was used to investigate relatedness. The sequence logo from groups of related clonotypes was used to identify the amino acid motifs.

### Study approval

This study received institutional ethical approval and all subjects provided written informed consent at Imperial College Healthcare NHS Trust.

## Results

### Subjects and sequencing data

Clinical, demographic, and biological sample information for the five relapsing–remitting MS patients and five IIH controls included in this study is shown in Table 1. The two groups were age and sex matched. All patients with MS met the McDonald diagnostic criteria,^13^ and subject MS‐4 had gadolinium enhancement on magnetic resonance imaging of the brain. There were no significant differences in PB or CSF white blood cell counts between MS patients and controls. Total numbers of TCR*β* in‐frame sequence reads were not significantly different between MS patients and controls for the PB and/or CSF compartments. On average, 4,445,954 reads were sequenced from the PB compartment and 3,768,585 reads were sequenced from the CSF compartment for each subject (Supplementary Section, Table S1).

**Table 1:** Demographic, clinical, and biological information

Subject^a^	Age (yr)	Sex	Diagnosis	EDSS score	Brain MRI	CSF IgG OCB	WBC in blood (10^3^/*μ*L)	CSF volume (mL)	WBC in CSF^b^
MS‐1	25	M	RRMS	2	MS	Negative	1.5	11	2.2 x 10^5^
MS‐2	29	F	RRMS	1.5	MS	Positive	2.0	11	2 x 10^5^
MS‐3	33	F	RRMS	1.5	MS	Positive	1.4	10	1.3 x 10^5^
MS‐4	28	F	RRMS	1	MS, with enhancement	Positive	1.4	11	1.3 x 10^5^
MS‐5	41	M	RRMS	6	MS	Positive	1.8	10	1 x 10^5^
IIH‐1	37	F	IIH	N/A	Normal	Negative	2.9	12	2 x 10^5^
IIH‐2	23	M	IIH	N/A	Normal	Negative	2.1	12	2 x 10^5^
IIH‐3	34	F	IIH	N/A	Normal	Negative	2.1	10	3.7 x 10^4^
IIH‐4	33	M	IIH	N/A	Normal	Negative	2.7	10	1.2 x 10^5^
IIH‐5	34	F	IIH	N/A	Normal	Negative	1.5	9	2 x 10^5^

EDSS, expanded disability status scale; MRI, magnetic resonance imaging; WBC, white blood cells; CSF, cerebrospinal fluid; OCB, oligoclonal bands.

a Characteristics of five multiple sclerosis (MS) patients and five idiopathic intracranial hypertension (IIH) controls are shown.

b Total number of cells used for TCR‐*β* repertoire analysis.

### Richness and diversity of the TCRβ repertoire are higher in peripheral blood compared with CSF

The statistically equivalent number of TCR*β* in‐frame reads between the PB and CSF (unpaired parametric T‐test, *P* = 0.41; Table S1) enabled us to compare repertoires across the two compartments with confidence. Our initial analysis evaluated the number of unique productive TCR*β* combinations (defined here as richness) between PB and CSF across all subjects. As shown in Figure 1A, richness in the PB compartment was significantly higher than in the CSF (*P* = 0.0019). Likewise, we also compared the level of T‐cell diversity, estimated as the number of unique TCR*β* clonotypes per sample normalized by cell count, between PB and CSF across all subjects. As with richness, diversity in the PB compartment was significantly higher compared with CSF (*P* = 0.013, Fig. 1B). However, the magnitude of this difference in diversity was less striking compared with the difference in richness, the latter presumably reflecting the higher numbers of T cells present in the PB samples.

### Diversity of the TCRβ repertoire is higher in the peripheral blood and CSF of MS patients compared with IIH controls

To estimate the complexity of the TCR*β* repertoire in MS patients compared with IIH controls, we evaluated clonotypic diversity in each immune compartment with respect to disease status. TCR*β* diversity was significantly greater in the PB and CSF of MS patients compared with IIH controls (Fig. 2A and B). The difference observed in the CSF may indicate the presence of a larger polyclonal repertoire in the intrathecal compartment of MS patients. As expected, the TCR*β* diversity was higher in the PB than the CSF.

### Greater clonal T‐cell expansions are present in the peripheral blood and CSF of MS patients compared with IIH controls

**Figure 3:**
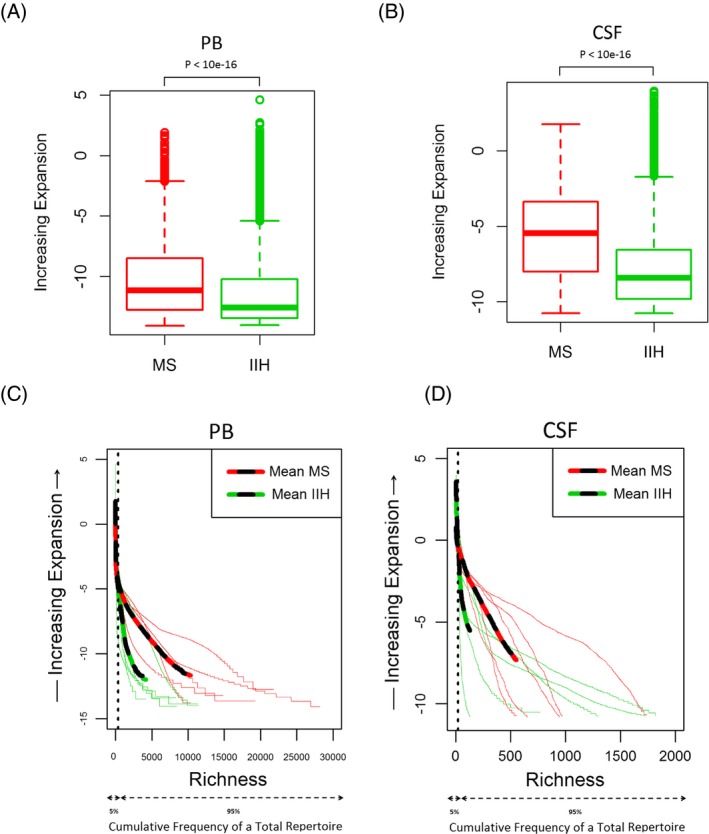
Expansion of TCR *β* clonotypes in the peripheral blood and cerebrospinal fluid (CSF) of multiple sclerosis (MS) patients and idiopathic intracranial hypertension (IIH) controls. Frequency of clonal expansion analysis by group samples in peripheral blood (PB) (**A)** and CSF (**B**). Frequency of clonal expansion analysis for each individual subject in PB (**C**) and CSF (**D**). Mean values: dashed red line for MS patients, dashed green line for IIH controls. Average frequencies: thick red line for MS patients, thick green line for IIH controls. TCR*,* T‐cell receptor.

As the level of TCR*β* diversity in a sample does not provide any direct information on the degree of clonal expansion, we examined the frequency of each individual clone from those five MS patients and five IIH controls. Expansion, defined here as the frequency of each unique TCR*β* sequence (i.e., clonotypes) in the total repertoire, was significantly higher in the PB and CSF of MS patients compared with IIH controls (log of % total clonotypes with *P* < 10^16^; Fig. 3A and B). Investigation of expansion by both disease cohort and compartment revealed that: (1) the number of unique TCR*β* sequences (clonotypes) in a repertoire with the greatest frequency would be represented by <5% of the total repertoire (left of the black dashed line; Fig. 3C and D); (2) the clonotypes with the highest frequency (top 5%) were insufficient to explain the differences in expansion observed between MS patients (red lines) and IIH controls (green lines); (3) the differences in expansion between MS patients and IIH controls were better explained by lower frequency clonotypes (right of the black dashed line; Fig. 3C and D); and (4) interpretation of expansion was clearer when the average frequencies for a sample group were used (represented by the thick red dashed lines for MS patients and green dashed lines for IIH controls; Fig. 3C and D). Significantly increased clonal T‐cell expansions were therefore present in the TCR*β* repertoire of MS patients compared with IIH controls.

### A nonshared (private) TCRβ repertoire exists in the CSF compartment compared to peripheral blood

**Figure 4:**
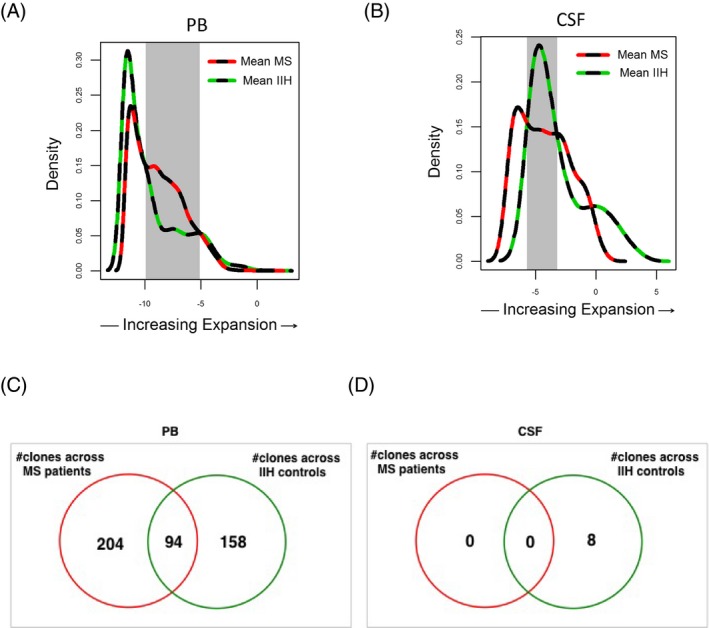
Distribution of expanded T‐cell clonotypes in the peripheral blood and cerebrospinal fluid (CSF) of multiple sclerosis (MS) patients and idiopathic intracranial hypertension (IIH) controls. Clonotype distribution as a function of expansion in the peripheral blood (PB) (**A**) and CSF (**B**) of MS patients (dashed red line) and IIH controls (dashed green line). Subsets of differentially expanded clones in PB (from −9.9 to −5.1 log frequency; gray shading) and CSF (from −5.7 to −3.2 log frequency; gray shading) were selected for phylogenetic tree analysis (Fig. 5). Number of shared clonotypes by CDR3 amino acid sequence across MS patients and IIH controls in PB (**C**) and CSF (**D**).

To evaluate the degree of expansion, defined as the range of frequencies of expanded TCR*β* clonotypes, and identify any differences in distribution between MS patients and IIH controls, the data were replotted as the average frequencies across clonotypes sorted by degree of expansion (Fig. 4A and B). Comparing the data by sample group and compartment, the range of expansion where differences exist between those five MS patients (red line) and five IIH controls (green line) becomes notably clear (shaded area; Fig. 4A and B). This observation allowed us to examine the V‐CDR3‐J sequences of individual clones within the frequency ranges that best separated subjects by disease status. We defined a clonotype as ‘shared’ if it was present in the PB or CSF of all subjects within a disease group. Comparing the V‐CDR3‐J recombinations of the shared clonotypes in PB across MS patients (*n* = 298) with the shared clonotypes in PB across IIH controls (*n* = 252), we found that a subset (*n* = 94) of these clonotypes were common (Fig. 4C). By contrast, no clonotypes were shared in the CSF among any of the five MS patients and only eight shared clonotypes were found in the CSF of IIH controls (Fig. 4D). Even though the probability of shared clones found in the PB compartment of MS patients and IIH controls was increased due the higher number of cells, these findings would indicate the presence of private TCR*β* repertoire expansions in the intrathecal compartment. All shared CDR3 sequences are shown in Table S2, S3 and S4.

### CDR3 sequence relatedness of expanded T‐cell clonotypes in the peripheral blood distinguishes MS patients from IIH controls

**Figure 5:**
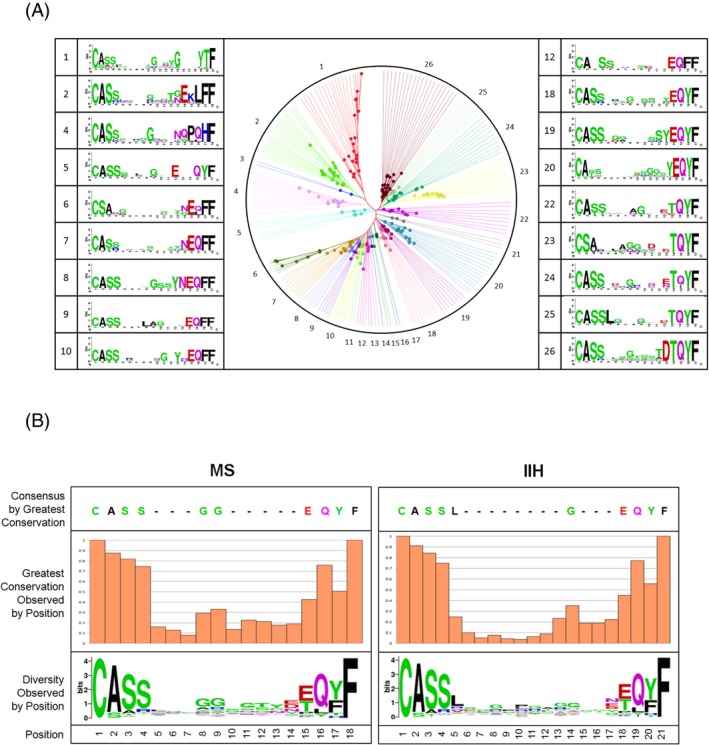
CDR3 sequence relatedness of T‐cell clonotypes expanded in the peripheral blood. Phylogenetic tree analysis of CDR3 amino acid sequence similarities in T‐cell clonotypes that were differentially expanded against idiopathic intracranial hypertension (IIH) and were shared in PB across all fivemultiple sclerosis (MS) patients (as shown in Fig. 4A, C). Representative CDR3 motif clusters are shown as sequence logos (**A**). CDR3 amino acid sequence logo from exclusively shared clonotypes across MS patients (*n* = 204) and IIH controls (*n* = 158) (**B**). PB, peripheral blood.

To explore the possibility that there may be a subset of clonotypes specific for MS patients, we analyzed the catalog of TCR*β* clonotypes expanded in PB for each patient group (Fig. 4A–C). As shown in Figure 4C, we identified 204 clonotypes in PB that were exclusively shared among all five MS patients. Likewise, 158 clonotypes were shared uniquely among all five IIH patients. Phylogenetic tree cluster analyses (CLC bio) with respect to CDR3 amino acid sequence similarities for each of these shared clonotypes were then performed to determine clonal relatedness. This analysis is shown for the 204 unique clonotypes in the PB of MS patients in Figure 5A. Twenty‐six clusters were identified that shared common CDR3 motifs by sequence similarity, and consensus sequence data for each cluster were visualized using web logos (Fig. 5A). Moreover, when all clusters were compared for relatedness, a consensus G (glycine) at positions eight and nine of the CDR3 loop emerged in the MS cohort that differed from the consensus sequence observed across the 158 shared clonotypes from IIH controls (Fig. 5B). These results suggest that TCR motifs may distinguish MS patients from controls in the PB. The corresponding analyses could not be performed for the CSF repertoires because no shared clonotypes were identified among MS patients (Fig. 4D).

### Compartmentalized expansion of T‐cell clonotypes in the CSF

**Figure 6:**
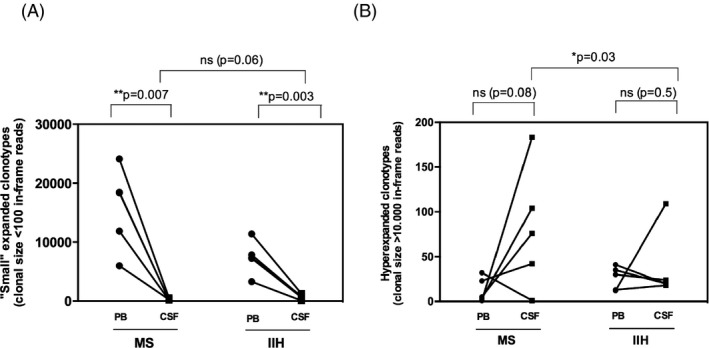
Numbers of unique TCR *β* clonotypes present at different degrees of expansion in the peripheral blood and CSF of MS patients and IIH controls. Analysis is shown for small expansion clonotypes, defined as clonal size <100 in‐frame reads (**A**), and hyperexpanded clonotypes, defined as clonal size >10,000 in‐frame reads (**B**). CSF, cerebrospinal fluid. IIH, idiopathic intracranial hypertension. MS, multiple sclerosis. TCR, T‐cell receptor.

As the overall frequency of expanded T cells was greater in the PB and CSF of MS patients compared with IIH controls (Fig. 3A and B), we asked whether this distribution would be mirrored by differences in the number of TCR*β* clonotypes categorized by “degree” of expansion. We classified two extreme groups of clonotypes: (1) those with <100 in‐frame reads (clonal size defined as “small expansion”); and (2) those with >10,000 in‐frame reads (clonal size defined as “hyperexpanded”). Significantly lower numbers of clonotypes in the small expansion group were observed in the CSF of MS patients (*P* = 0.007) and IIH controls (*P* = 0.003) relative to PB, whereas no significant difference was observed between disease cohorts with respect to CSF (Fig. 6A). Higher numbers of hyperexpanded clonotypes were observed in the CSF of MS patients relative to PB, which did not reach statistical significance most likely due to a single outlier (*P* = 0.08), whereas this trend was not observed in IIH controls (Fig. 6B). However, a significant difference was observed in the CSF between MS patients and IIH controls (*P* = 0.03). These observations are consistent with a disease‐specific enrichment of highly expanded clonotypes in the CSF of MS patients.

To determine whether hyperexpanded clonotypes in the CSF of MS patients were also present in the PB (Fig. 6B), we examined the number of shared CDR3 amino acid sequences by compartment for each subject (Table 2). Very few hyperexpanded clonotypes in the CSF were found in PB across either of the disease groups (average 2.06% of total sequences in CSF). Moreover, only a single clonotype (from >10,000 clones) identified in the CSF of all subjects (V4.3‐CASSQRLAGSTDTQYF‐J2.3) was also present in PB. The rarity of these public clonotypes and the low frequency of any shared sequences between anatomical sites support the interpretation that the vast majority of T‐cell clonotypes detectable in the CSF are compartmentalized to the intrathecal space.

**Table 2:** Hyperexpanded T‐cell clonotypes (clonal size >10,000 in‐frame reads) shared between PB and CSF of MS patients and IIH controls

Subject	Total sequences in PB	Total sequences in CSF	Number of shared clones CSF (n/total)	Shared sequences (% CSF repertoire)	Clone V‐CDR3‐J
MS‐1	72,414	1,757,415	0/105	0%	–
MS‐2	342,597	700,041	4/43	0.1%	V7.2‐CASSPGYSGYEQYF‐J2.7 V7.6‐CASSLEPGRNEKLF‐J1.4 V7.2‐CASSLGGGISYEQYF‐J2.7 V7.2‐CASSPLAGGTYNEQF‐J2.1
MS‐3	51,234	4,036,432	1/185	2.59%	V4.1‐CASSQDRYRQNTEAF‐J1.1
MS‐4	244,397	2,202,176	0/75	0%	–
MS‐5^a^	9,631,112	11,386	–	–	–
IIH‐1	834,970	985,623	1/20	3.7%	V12.3‐CASQQGSGSYEQYF‐J2.7
IIH‐2	399,951	3,085,051	0/24	0%	–
IIH‐3	294,631	553,871	0/19	0%	–
IIH‐4	2,737,399	914,804	0/22	0%	–
IIH‐5	336,773	3,694,466	1/109	9.2%	V7.2‐CASSLGAGGSTQYF‐J2.3

PB, peripheral blood.

a Subject MS‐5 had one T‐cell clonotype classified as hyperexpanded in the CSF compartment.

## Discussion

Immune cell entry into the CNS is tightly controlled under physiological conditions and immune surveillance is localized to the perivascular and subarachnoid spaces (part of the CSF compartment).^14^ Trafficking of T lymphocytes across the blood–brain or blood–CSF barrier is known to play a crucial role in the initiation of an inflammatory response in the CNS of patients with MS and is a major therapeutic target in this disorder. Although there are reports of biased TCR*β* chain repertoire profiles in the CSF of patients with MS,^7^, ^15^, ^16^ a lack of sequencing depth and clonal skewing due to in vitro expansion potentially complicate the interpretation of these data. To avoid these pitfalls, we used an unbiased molecular technique to amplify and deep sequence TCR*β* rearrangements present in unsorted T cells isolated from the PB and CSF of relapsing–remitting MS patients and IIH controls. It is notable that IIH is widely regarded as a disorder resulting from abnormalities in CSF volume homeostasis. Chronic inflammation has been implicated in this condition,^11^ and CSF oligoclonal bands (OCBs) were recently identified in 30% of cases.^17^ However, no serological or CSF indices of inflammation were detected in the IIH controls studied here.

Although TCR*β* repertoire diversity was higher in both the PB and CSF of MS patients compared with IIH controls, expanded clonotypes were significantly more frequent across both compartments in MS patients. In the frequency ranges best differentiating MS and IIH, few clonotypes were shared among all subjects in PB and none was shared in CSF. Phylogenetic tree analysis of the shared clonotypes from PB revealed potentially distinctive CDR3 motifs in MS patients.

Our analyses of the dataset first examined richness, defined as the absolute number of unique TCR*β* clonotypes,^18^ and diversity, defined as the fraction of unique clonotypes in a sample normalized by cell count, in PB and CSF. Richness of the TCR*β* repertoire was much lower in CSF compared with PB, likely explained by the higher number of T cells obtained from unsorted PB samples compared with CSF (approx. 5 × 10^5^ T cells in PB vs. 1 × 10^5^ T cells in CSF). However, the number of input cells was used to normalize TCR*β* repertoire diversity by applying Shannon's entropy algorithm, and the number of total in‐frame reads were not significantly different between groups. We therefore felt confident comparing repertoire diversity by compartment and subject. In addition, we applied the Leave‐One‐Out statistical test to reinforce the significant *p*‐value between our groups of five subjects. Diversity of the TCR*β* repertoire was lower in CSF compared with PB in all subjects. A recent study using a different molecular approach (multiplex PCR for library preparation) reported a highly diverse TCR*β* repertoire in the PB and CSF of MS patients and controls with other neurological disorders.^19^ By contrast, we found greater TCR*β* diversity in the PB and CSF of MS patients compared with IIH controls. Different methodologies and patient cohorts may underlie the diverging results. Using deep sequencing of the TCR*β* repertoire across four different groups of healthy donors, Britanova et al. demonstrated that repertoire diversity decreased significantly with age.^20^ Another recent study also demonstrated a decline in TCR repertoire diversity linked to the number of naïve T cells in elderly subjects.^18^ As the higher TCR repertoire diversity observed in the PB and CSF of MS patients compared with IIH controls cannot be explained by age differences in our subject groups, we suggest that this difference reflects a dysregulated T‐cell response associated with MS. Clearly, these proof‐of‐principle observations need to be extended to a large cohort of MS patients and controls.

The analysis of clonotype frequency revealed significantly higher mean clonal frequencies in MS patients compared with IIH controls across both compartments, albeit with a more prominent difference in CSF. The greater diversity of PB and CSF repertoires in MS patients, discussed above, reassures us against the possibility that these higher clonal frequencies may be due to sampling error (e.g., from lower numbers of input cells). By analyzing the cumulative frequency curves, we were able to delineate the range of frequencies that contributed most to the observed differences. Through a density plot representation, we were then able to define a specific range of clonal expansion that revealed differences in the number of clonotypes between MS patients and IIH controls in both PB and CSF. This analysis allowed us to more selectively target a search for the presence of shared T‐cell clonotypes 21 within compartments and between disease groups. Although we identified a robust number of shared clonotypes in the PB across all five MS patients (*n* = 204) as well as in all five controls (*n* = 158), very few shared clonotypes were observed in the CSF across IIH controls, and no clonotypes were common in the CSF across all MS patients. These results suggest that the CSF compartment may have a more private or “nonshared” TCR repertoire compared with PB. Accordingly, pathogenic T‐cell responses may be driven by individual clonotypes specific for a variety of antigen(s) present solely in the CNS. Recently, sharing of predominant T‐cell clones between CNS lesions, CSF, and blood CD8^+^ T cells was found in three MS patients when the TCR *β*‐chain repertoire was analyzed using a multiplex amplification approach for V‐ J genes.^22^ Clonally infiltrating, expanded CD8^+^ and CD4^+^ T‐cell in demyelinating MS lesions pattern II were also identified in the CSF compartment of MS patients after ex vivo culture expansion.^23^

The phylogenetic tree analysis of “public” T‐cell clonotypes found exclusively in the PB of MS patients revealed a group of 26 clusters, which showed amino acid sequence relatedness across the CDR3 loop. Although the antigen specificity of these TCRs remains unknown, we found a consensus motif with a conserved glycine (G) residue at CDR3 positions eight and nine in MS patients that was not conserved in IIH controls. Further studies are needed to determine whether this motif constitutes an MS‐specific signature and potential biomarker of disease in the peripheral TCR*β* repertoire.

After investigating the clones present at different density in the intermediate‐ to high‐frequency ranges in MS and controls and their relatedness, we focused our attention on “hyperexpanded” versus “small” expanded clonotypes. While the number of small clonotypes was significantly greater in PB than in CSF, both in MS and in IIH, and did not differ in the CSF between MS and IIH (Fig. 6A), there was a clear trend toward a higher number of hyperexpanded clonotypes in the MS patients’ CSF compared with that in the PB; and the number was significantly higher than that in controls (Fig. 6B). We suggest that these results reflect a disproportional clonal expansion of T cells in the CSF of MS patients. As very few of these hyperexpanded clones observed in the CSF were also present in the PB compartment, the data support the notion of a strongly compartmentalized intrathecal expansion of T cells in MS.

In conclusion, we used an unbiased method for TCR repertoire analysis to demonstrate clonal T‐cell expansions in the CSF of MS patients. The detection of highly expanded private clonotypes in the intrathecal compartment is consistent with the possibility of disease‐inducing T‐cell activation by CNS‐resident stimuli and signals. Although the antigen specificity of these clonotypes remains unknown, our analysis will inform the selection of relevant TCR*β* sequences for such downstream studies.

## Author contributions

Alessandra de Paula A. Sousa performed research, analyzed data, and wrote the manuscript; Kory R. Johnson analyzed data and wrote the manuscript; Richard Nicholas designed research; Sam Darko contributed for the TCR bioinformatics data analysis; David A. Price discussed the data; Daniel C. Douek contributed for the TCR molecular technique; Steven Jacobson designed research and wrote the manuscript; and Paolo Muraro designed research and wrote the manuscript.

## Conflict of Interest

Dr. De Paula Alves Sousa reports grants from the National Multiple Sclerosis Society. Dr. Nicholas reports grants, personal fees, and non‐financial support from Biogen, personal fees from Genzyme, personal fees and non‐financial support from Novartis, personal fees from Roche, outside the submitted work. Dr. Price reports grants from WELLCOME TRUST, NHMRC, outside the submitted work. Dr. Muraro reports grants from UK MS Society, during the conduct of the study; personal fees and non‐financial support from Merck Serono, personal fees from Biogen, personal fees and non‐financial support from Buyer, personal fees from Novartis, outside the submitted work.

## Supporting information


**Table S1.** High‐throughput sequencing dataset of TCR*β* repertoire in the PB and CSF compartment of MS patients and IIH controls.
**Table S2.** CDR3 amino acid sequences (clonotypes) shared among the five IIH controls in the CSF compartment.
**Table S3.** CDR3 amino acid sequences (clonotypes) shared among the five MS patients in the PB compartment. By phylogenetic analysis, the clonotypes sequence‐relatedness was investigated.
**Table S4**. CDR3 amino acid sequences (clonotypes) shared among the five IIH controls in the PB compartment.
