# Supplementary material for: Intrathecal T‐cell clonal expansions in patients with multiple sclerosis
Source: Ann Clin Transl Neurol. 2016 Apr 20;3(6):422–33. doi: 10.1002/acn3.310 (PMC4891996; doi:10.1002/acn3.310)
Supplement: Supplementary file 1 — Table S1. High‐throughput sequencing dataset of TCRβ repertoire in the PB and CSF compartment of MS patients and IIH controls. Table S2. CDR3 amino acid sequences (clonotypes) shared among the five IIH controls in the CSF compartment. Table S3. CDR3 amino acid sequences (clonotypes) shared among the five MS patients in the PB compartment. By phylogenetic analysis, the clonotypes sequence‐relatedness was investigated. Table S4. CDR3 amino acid sequences (clonotypes) shared among the five IIH controls in the PB compartment. [file ACN3-3-422-s001.pdf]

Supplementary Section

Table S1

Subject	Sample	Number of total reads	TCR in-frame reads	In-frame (%)	Richness	Shannon Diversity
MS-1	CSF	4,716,699	3,403,760	72	1009	0.49
	PB	2,874,781	1,889,915	65	15,641	0.59
MS-2	CSF	1,727,277	1,340,061	77	562	0.41
	PB	3,705,066	2,498,859	67	19,937	0.50
MS-3	CSF	8,211,199	5,602,060	68	992	0.49
	PB	1,787,395	1,376,469	77	22,302	0.64
MS-4	CSF	3,796,044	2,630,387	69	677	0.40
	PB	5,899,141	4,210,771	71	10,908	0.58
MS-5	CSF	1,482,178	1,128,776	76	1,800	0.58
	PB	7,808,614	5,169,968	66	28,877	0.56
IIH-1	CSF	2,500,116	1,544,754	61	1,324	0.36
	PB	3,253,121	2,121,313	65	8,540	0.40
IIH-2	CSF	4,513,018	3,143,787	69	129	0.22
	PB	3,310,342	2,267,938	68	4,370	0.46
IIH-3	CSF	1,707,293	727,561	42	778	0.34
	PB	4,373,604	3,328,922	76	8,611	0.49
IIH-4	CSF	2,394,998	1,200,920	50	1,878	0.35
	PB	4,749,025	2,979,790	62	11,567	0.26
IIH-5	CSF	6,637,032	4,469,764	67	1,774	0.42
	PB	6,698,455	4,804,131	71	11,996	0.57

N
o statistic differences was found in comparison of the total numbers of TCRβ sequencing in-frame reads between PB (P=0.93) and CSF (P=0.58) counts of MS patients and IIHcontrols (unpaired T-test with Mann-Whitney correction) and between PB and CSF counts for all subjects (parametric paired T-test, P=0.47). For the analysis of Richness and Shannon Diversity data please see Fig.1.

Table S2

1. CASSTYNEQFF
2. CSARNGLPDETQYF
3. CASRGGARGQETQYF
4. CASSLHTRVQETQYF
5. CASSPGTSYEQYF
6. CASSQRLAGSTDTQYF
7. CASSSLAGYEQYF
8. CASSYSLADSYEQYF

Table S3

1. CASSSGLGGYEQYF
2. CASSSYNEQFF
3. CSAIAGGAGSYEQYF
4. CASSLNSGRITSEQYF
5. CASTLAGGTYEQYF
6. CATSDPGQGRGETQYF
7. CASSLFNPEEETQYF
8. CASHEDGNTIYF
9. CASKIQGGGSPLHF
10. CASSLGLAGGTDTQYF
11. CASSALVGSGRAKNEQFF
12. CASSNQQGANEKLFF
13. CASGSYGTSGGEQYF
14. CASSTGDATNEKLFF
15. CASSVGGGASGMYNEQFF
16.CATSDLATSGRGEQFF
17. CASTAMQAIRGTQYF
18. CASSLEFPGTGGGEQYF
19. CASSFVTVRESYTF
20. CASSDRGGNQPQHF
21.CASRSRQFSEQYF
22. CASTTGRSSYEQYF
23. CASSQDSTDTQYF
24. CASGDRGANTGELFF
25. CATSDLSLDYNEQFF
26. CASSYAPGDTQYF
27. CASSEDGARVLGNTIYF
28. CASSSVAGGQPQHF
29. CASSLGRVFGSEQYF
30. CASSWDRRGTQYF
31. CASSFGGSYEQYF
32. CASSPRRQGANYGYTF
33. CASSRVGTDTQYF
34. CASSEASGNDEQFF
35. CASSQETETQYF
36.CASDRDRVNSPLHF
37.CASRISGSGTGELFF
38. CASSERLTDEQFF
39. CASRRSGRSQETQYF
40.CASSQELAGVADTQYF
41. CASSRQINYGYTF
42. CASSPGGKGGNQPQHF
43. CAISDGDRPYSYEQYF
44. CSAPLAGGTDTQYF
45. CASSLGGARGYTF
46. CASSFYSGAGSFF
47. CSAGQGETQYF
48. CSDSTGDGYGYTF
49.CASRETGFEQFF
50. CASSQTDGYTF
51. CSATDSYGYTF
52. CASSLAGTLNEQFF
53. CASSGLGSSERTDTQYF
54.CSARRAQGSDTQYF
55. CASSFSWGQETQYF
56.CASSSHDLAGGYSEQFF

57. CASRRTPPPGQGSEKLFF
58. CASRDSSTDTQYF
59. CASSQENEKLFF
60. CASSVRRTDEQFF
61. CASSSPGRSGYTF
62. CATSDSGTAYEQYF
63. CASSSGQLTISAQHF
64. CASSLGQGPNQPQHF
65. CASSLTERGRTDTQYF
66. CASSLAGVGNEQFF
67. CASSQRVASSGTYNEQFF
68. CASRPGGAADTQYF
69.CASSSGRTGSGYNEQFF
70. CASSQDEGQFHSYEQYF
71. CSAVRAHGYTF
72. CASSAQGYEQYF
73. CASSDEQGRGYTQYF
74. CARPLSGGAGGELFF
75. CASSTVAVHEQYF
76. CASKRRDGTGELFF
77. CASSLSGYNEQFF
78. CASSYLTSGSADTQYF
79. CSARGLAGGTDTQYF
80. CASSLDRRASGNTIYF
81. CASSWGLAGLGNEQFF
82. CASSSTGTSTTDTQYF
83. CASSLSGQLAYTF
84. CASSLAGQGSNEKLFF
85. CASSGTSGPYHEQFF
86. CASSQDRYRTNEKLFF
87. CASSPYFLGSSYNEQFF
88. CAPWEGPGVHYGYTF
89. CASTPAGGTGGGYTF
90. CASSLAGGSTGELFF
91. CSASGSQSSYNEQFF
92. CASSLPSRGNEQFF
93. CASSTYNEQFF
94. CASSLHTRVQETQYF
95. CASSLEPISNEQFF
96. CASSSSENRTNYGYTF
97. CASSLDGTERSYEQYF
98. CASSLSYGGANYGYTF
99. CASSYDRDTGELFF
100. CSARDAGGSDRETQYF
101. CASSFAGGGEQYF
102. CASRQTWHNEQFF
103. CATMGSGLSYEQYF
104. CASSFRVQETQYF
105. CASSLLGQINEQYF
106. CASSTTPGLSQETQYF
107. CASSYRPQNYPEQFF
108. CASGSGTGGAHEDTQYF
109. CSVLGQGSGANVLTF
110. CASRSILITYNEQFF
111. CASSLGPGGPDTQYF
112. CASSPPFSRLGEQFF
113. CASSRTGAGELFF
114. CASSSWGENDEQFF
115. CASSLISQLYEQYF

116. CASRGTRGEQYF
117. CASSPTSGPSSYEQYF
118. CAGSTGPYEQYF
119. CASSQGASGWSSYEQYF
120. CSARGRTGDTGELFF
121. CASRTGLAGNNEQFF
122. CASSLMDSNQPQHF
123. CATSDPGTSGFYEQYF
124. CASSSGLARQETQYF
125. CASSSRGTTDTQYF
126. CASSSFSSYEQYF
127. CASSVDRGQETQYF
128. CASSLSPGAGETQYF
129. CASIGTAYTGELFF
130. CASSPGQGGYTF
131. CASSLRQGAYNEQFF
132. CASSSRDKRHEQFF
133. CASSRDGESSYEQYF
134. CASSLGGREQFF
135. CASSQAIQGGGDTQYF
136. CASSRKQGLQETQYF
137. CASSLGVGGRIEQYF
138. CASSGQQETQYF
139. CASSLSSGTITDTQYF
140. CASSQRTGSEQYF
141. CASMGLANTGELFF
142. CASTFGAGDYEQYF
143. CASSLSETGELFF
144. CASSRQGMNTGELFF
145. CASSYSLADSYEQYF
146. CASSVGYNEQFF
147. CAWSVRGYEQYF
148. CASSLGYQGPTDTQYF
149. CASREGAYNEQFF
150. CSVEVGQGREQYF
151. CASSQDGAGLTGELFF
152. CSARGHTGVAGELFF
153. CATSRPGQGVQTQYF
154. CASRGGARGQETQYF
155. CASSPGTSYEQYF
156. CASSLEGRAPQPQHF
157. CSANMVGGNYNEQFF
158. CASSAYDRGTEQFF
159. CASSTGTGTTDTQYF
160. CSVEGLATFSTQYF
161. CASSSEQNQPQHF
162. CASRIEGSSYNEQFF
163. CASSQRLAGSTDTQYF
164. CASSKDWGGSSYNEQFF
165. CSAPTSGGPDTQYF
166. CASSPRQGHEQFF
167. CASSSLAGYEQYF
168. CASSQITLTSGISEQYF
169. CSAQRRQIDTQYF
170. CASSAPDSSYEQYF
171. CAISDHLRSYEQYF
172. CSALQGASTDTQYF
173. CASSLTGRGYGYTF
174. CAISDLRQGRQDEQFF

175. CASSFTDTQYF
176. CASSFSHSYEQYF
177. CSAARTVETQYF
178. CSVEDPGTSANSYNEQFF
179. CASSQAEDLVEQYF
180. CASSELASTPGTDTQYF
181. CASSPTGYEQYF
182. CASSLEPGRNEKLFF
183. CASSLGGGISYEQYF
184. CASSLGGFATQYF
185. CASSPLAGGTYNEQFF
186. CASSPGYSGYEQYF
187. CASTPVFGSWDGYTF
188. CASSQDHYGGRTNEQFF
189. CASSSGGASTDTQYF
190. CASSPGAGMKETQYF
191. CASTSPAGSGGPRSSYNEQFF
192. CASSLGQGQYNEQFF
193. CASSLGVSGSSYNEQFF
194. CASSLGGPRDTQYF
195. CASSFKSVASSTDTQYF
196. CASSQVEGQETQYF
197. CASSSQGRDNRQPQHF
198. CSARGSEDTGELFF
199. CSASPQINEQFF
200. CASSEGAENQPQHF
201. CASSAGSPWANGNYGYTF
202. CASSPQTGTGYGYTF
203. CASSQDRYRQNTEAFF
204. CASSLRENYGYTF
205. CASSPTRGFYNEQFF
206. CASSSKGTGLAEYEQYF
207. CASSLVPTSGGVVAEQFF
208. CASSLSGASYEQYF
209. CASSHRVGVSYEQYF
210. CASSSRTGVHSNQPQHF
211. CASSFQLSGSSYNEQFF
212. CASSELTSEKLFF
213. CASSESDLGSTDTQYF
214. CASRVRGANTGELFF
215. CASSQELGTGGRGYTF
216. CASRPTTFQETQYF
217. CSARVYSGRGVVSGRDGEQFF
218. CASSAGPGGYGYTF
219. CASSANMGTLTDTQYF
220. CASSFPSLAGVSYNEQFF
221. CASRPRGLADERGTQYF
222. CASRLSGSSYNEQFF
223. CASSLNLAGGHYNNEQFF
224. CASSYGWSSYNEQFF
225. CASSPTVHTDTQYF
226. CASSLSASGSFLTGEQYF
227. CAILIGWQQFF
228. CASSEEAQWVYEQYF
229. CSARFAGTTYNEQFF
230. CSATGLLASYEQYF
231. CASSVYRVPGEKLFF
232. CASSLAGVAGDYNEQFF
233. CASSPGTGEGYEQFF

234. CASSLVGREQYF
235. CASSGHSNQPQHF
236. CASTLGQGIFYEQYF
237. CASSWGGGDQPQHF
238. CASRLIDRRNQPQHF
239. CASSYSDWTLEPQHF
240. CASSSWTDSYEQYF
241. CASSPASGGFYNEQFF
242. CSVPLFTGELFF
243. CSVEEGQTTDTQYF
244. CASSLSGQRVYEQYF
245. CASSLGGQGVLSDGYTF
246. CASSLMSGKREEEYF
247.CASRNDPSGDEQFF
248. CASDRAGGSGELFF
249. CASRPGLAAYNEQFF
250. CASSLVGLEQYF
251. CASSFWTSGSNEQFF
252. CASSTSGGSYNEQFF
253. CASSFPGVHEQYF
254. CASSLLDLSGANVLTF
255. CASSQALGLAGGEQYF
256. CASSRQSGLSYEQYF
257. CASSQAGGGYNEQFF
258. CASSPRGFRGPGEKLFF
259. CASSARYEQYF
260. CASSLDPGRLTYEQYF
261. CASSLLDLSGANGLTF
262. CASSFPGLNEQYF
263. CASSLRNSETQYF
264. CASRPQGRSSTDTQYF
265. CASTLAGQETQYF
266. CSARAAGPLGFLSLDYTQYF
267. CSARDVGGLSGTQYF
268. CASSQWGQSADTQYF
269. CASSLVGSSTDTQYF
270. CASSSNGNEQFF
271. CASSYGGPGDEQFF
272. CSVDGTSGNTDTQYF
273. CSVEWVVNYGYTF
274. CASRPSDRGTYEQFF
275. CASSYSQGTADTQYF
276. CATSRDGTSGGDTQYF
277. CASSAGTGYTF
278. CASQQGSGSYEQYF
279. CASSLALDWEQFF
280. CSARNGLPDETQYF
281. CASSSNLAGGNSYEQYF
282. CASSLGAGGSTQYF
283. CASSLKDTQYF
284. CATRDTDGLAGGYEQYF
285. CASSHPPGGTEQYF
286. CASSVDGTYGYTF
287. CASSSRLAGVGETQYF
288. CASSSLPPKVNEQFF
289. CASSEAGPLSGANVLTF
290. CSALGQGAYNEQFF
291. CASSPGPGNEQFF
292. CASSLGQPYGYTF

293. CASRKGQGANQPQHF
294. CASSSLADSYNEQFF
295. CASSPRGWEGSYNEQFF
296. CASSQALGTQYF
297. CASSFGTSNTDTQYF
298. CASSFRGEGYEQYF

Table S4

1. CARPLSGGAGGELFF
2. CASSSTGTSTTDTQYF
3. CASSTYNEQFF
4. CASSLHTRVQETQYF
5. CASIGTAYTGELFF
6. CASSSRDKRHEQFF
7. CASSQAIQGGGDTQYF
8. CASSYSLADSYEQYF
9. CASSVGYNEQFF
10. CASSLGYQGPTDTQYF
11. CASREGAYNEQFF
12. CSVEVGQGREQYF
13. CASSQDGAGLTGELFF
14. CASRGGARGQETQYF
15. CASSPGTSYEQYF
16. CASSLEGRAPQPQHF
17. CASSQRLAGSTDTQYF
18. CASSKDWGGSSYNEQFF
19. CASSSLAGYEQYF
20. CASSPGYSGYEQYF
21. CASTSPAGSGGPRSSYNEQFF
22. CASSLRENYGYTF
23. CASSPTRGFYNEQFF
24. CASSLVPTSGGVVAEQFF
25. CASSELTSEKLFF
26. CAISDPTGQGGIYEQYF
27. CASSRTPASGRADTQYF
28. CSARHLGDGYTF
29. CASSLMGAQPQHF
30. CASSQELGTGGRGYTF
31. CASSFSVLTDTQYF

32. CSASPTGGEQYF
33. CSASRSGWATGLDQPQHF
34. CASSSTGSGEQYF
35. CASSSPLGSLTDTQYF
36. CASSRQGNGYTF
37. CASSLASGRSYEQYF
38. CASSQQIGARLSYNEQFF
39. CASSLGAGLGEKLFF
40. CASSLIQINTIYF
41. CSASVTWGLLPFSGNTIYF
42. CASSVDGQGYEQYF
43. CASSLGVQNGELFF
44. CASSSDRDEQFF
45. CASSTGTENYGYTF
46. CASSGTGDSYNEQFF
47. CASSPQGAETQYF
48. CASRPTTFQETQYF
49. CASSSVPNNEQFF
50. CASSQPRGQGAASGPNEQFF
51. CASSLAPRDEDEQFF
52. CASSLAGLYEQFF
53. CSARVYSGRGVVSGRDGEQFF
54. CASSLYGGSWETQYF
55. CASSLMGGGGPVGQYF
56. CASSSKQGWYEQYF
57. CASIGGRGGLTQYF
58. CSANPSTSGPPDTQYF
59. CASSRYRETNEKLFF
60. CASSPTKTGELFF
61. CASSGGTGNQPQHF
62. CASSVEPLAVPREQYF
63. CASGGWREEGETQYF
64. CASRETQGALHEQFF
65. CASSAGPGGYGYTF
66. CASSLSMWDEQYF
67. CASSAIGGITDTQYF
68. CSASPVERDLYEQYF
69. CASSLADRPAYEQYF
70. CASSDTTSYEQYF
71. CATSRLRTGSGDEQYF
72. CASRLRVGNEKLFF
73. CASSARGQATYEQYF
74. CASSSPIEGGTDTQYF
75. CASSVYGTSRKGEQYF
76. CASSLAGGNHNEQFF
77. CASSLGQGISGNTIYF
78. CASRLTGGGNQPQHF
79. CAISEGQGAETQYF
80. CSASQGAVTDTQYF
81. CASTTWGDSNSPLHF
82. CASSLPQGKNEQYF
83. CASSLVTGAGNQPQHF
84. CASSPLAGIYEQFF
85. CASSLRRGGMETQYF
86. CASSSLYRAQGTQYF
87. CASSLAGTKTGNTIYF
88. CASSQLRGSTNTGELFF
89. CASSANMGTLTDTQYF
90. CASSFPSLAGVSYNEQFF

91. CAINIGTGRQETQYF
92. CASSQEWRGYSYEQYF
93. CASSSGTGGADTQYF
94. CASSQDPPSGANTGELFF
95. CASSSGAGQGNEQYF
96. CASSQREGTSGKKQFF
97. CASSQALGQTFSGNTIYF
98. CASSLLAGVADTQYF
99. CASRPRGLADERGTQYF
100. CASSFSGDSYNEQFF
101. CASSVRTSGSTGELFF
102. CASSPVTGTNTGELFF
103. CASSLWDRVIYNEQFF
104. CASRGRVVQETQYF
105. CASSSPELAGGHEQYF
106. CASSLGSMREGFYNEQFF
107. CATSRDRGGWYGYTF
108. CASSLAGAPYNEQFF
109. CASSLEADSYNEQFF
110. CASSSQREGIGELFF
111. CASSRLAGVYEQYF
112. CASRLSGSSYNEQFF
113. CASSLLTSGNTGELFF
114. CASSLNLAGGHYNNEQFF
115. CASSYQVRDSYNEQFF
116. CASSYGWSSYNEQFF
117. CASNLQGKGSPLHF
118. CASSSISGRAIETQYF
119. CASSLVPGTGEGETQYF
120. CASSPVRAVAEKLFF
121. CASSSRRRETTKETQYF
122. CASSQNFRSAQYF
123. CSAEEGTDTQYF
124. CASSFLGGSGYTF
125. CASSSLKGSGRPDTQYF
126. CASSPTVHTDTQYF
127. CASSLRRGNNEQFF
128. CASSLGTGQETQYF
129. CASSLSASGSFLTGEQYF
130. CAILIGWQQFF
131. CASSLIGTGTETQYF
132. CASSESGTIGETQYF
133. CASSLEPAKNIQYF
134. CASSPFREVDNEQFF
135. CASRPSGGRAGNEQFF
136. CASSEEAQWVYEQYF
137. CSARFAGTTYNEQFF
138. CASSSGTGGSGNIQYF
139. CASSPRGLAGGSETQYF
140. CASSSPDYNEQFF
141. CSATGLLASYEQYF
142. CASSPLSGEQYF
143. CATSDFYSGFYEQFF
144. CASSVYRVPGEKLFF
145. CASSSVAGDGTDTQYF
146. CASSLWTGIEQFF
147. CASSQESWQYSYNEQFF
148. CASRHELGTGGTNPVYNEQFF
149. CASSLARTSGSMETQYF

150. CASSLGLAGHDNEQFF
151. CASSLAGVAGDYNEQFF
152. CASRKDRGLSPYTDTQYF
153. CATSRAKGGGSSYEQYF
154. CASSEGPIIEQFF
155. CSAKRDRTDTQYF
156. CASSLEGRGQETQYF
157. CASSVTGTGYGYTF
158. CSATLREPYEQYF
159. CASTLQGKGTQYF
160. CAISDPGPGNTIYF
161. CASSLFDVPTGELFF
162. CASSLSGYEQYF
163. CASSWDRTEGYEQYF
164. CASSPSGRASYEQYF
165. CATSREGGETQYF
166. CASKYGRTSLSRETQYF
167. CASSSHRSSYEQYF
168. CATSRDRGYEQYF
169. CASSLVWTDTQYF
170. CASSWGGLEPNEQFF
171. CSARVVAGEGKKDTQYF
172. CASTLDRVYEDTQYF
173. CASSEPGGSNEQFF
174. CASSRTSGTLYNEQFF
175. CASSLVGREQYF
176. CASSLAGGSPTGELFF
177. CASSGHSNQPQHF
178. CASTLGQGIFYEQYF
179. CASSWGGGDQPQHF
180. CASRLIDRRNQPQHF
181. CASSYSDWTLEPQHF
182. CASSSWTDSYEQYF
183. CASSPASGGFYNEQFF
184. CASSLVVSEQFF
185. CSVPLFTGELFF
186. CSVEEGQTTDTQYF
187. CASSLSGQRVYEQYF
188. CASSLGGQGVLSDGYTF
189. CASSLMSGKREEEYF
190. CASRNDPSGDEQFF
191. CASDRAGGSGELFF
192. CASRPGLAAYNEQFF
193. CASSSTGELFF
194. CASSLVGLEQYF
195. CASSFWTSGSNEQFF
196. CASSSGTGPKQPQHF
197. CASSFPGVHEQYF
198. CASSLLDLSGANVLTF
199. CASSQALGLAGGEQYF
200. CASSRQSGLSYEQYF
201. CASSQAGGGYNEQFF
202. CASSPRGFRGPGEKLFF
203. CASSARYEQYF
204. CASSLDPGRLTYEQYF
205. CASSLLDLSGANGLTF
206. CASSFPGLNEQYF
207. CASSLEGGTDTQYF
208. CASRATPAGHGYTF

209. CASRSSGDTDTQYF
210. CASSRPGQGDTEAFF
211. CSARDRGLTYEQYF
212. CASARGGNQPQHF
213. CASSQDWGASGGVGETQYF
214. CASRPQGRSSTDTQYF
215. CASSSRDARETQYF
216. CASTLAGQETQYF
217. CSARAAGPLGFLSLDYTQYF
218. CSARDVGGLSGTQYF
219. CASSEPGLAEETQYF
220. CSAPGQGTDTQYF
221. CASSQWGQSADTQYF
222. CASSQEIAGSYTF
223. CASSLVGSSTDTQYF
224. CASRTGAATNEKLFF
225. CASRPEQRSNQPQHF
226. CASSSNGNEQFF
227. CASSYGGPGDEQFF
228. CASSERGSQETQYF
229. CASSLERQHQETQYF
230. CASSGPGTPSETQYF
231. CASSLSPGTGYEQYF
232. CASSLYKGENEQFF
233. CASRPSDRGTYEQFF
234. CSVESGTSAYNEQFF
235. CATSKLQAVSGELFF
236. CASSAGTGYTF
237. CASQQGSGSYEQYF
238. CSARNGLPDETQYF
239. CASSSGNTIYF
240. CASDQGVNSEQYF
241. CASSLKDTQYF
242. CATRDTDGLAGGYEQYF
243. CASSHPPGGTEQYF
244. CASSVDGTYGYTF
245. CASSSLPPKVNEQFF
246. CASSEAGPLSGANGLTF
247. CASSEAGPLSGANVLTF
248. CASSPGPGNEQFF
249. CASSLGQPYGYTF
250. CASSSLADSYNEQFF
251. CASSPRGWEGSYNEQFF
252. CASSQALGTQYF
